# Occurrence of *Fusarium* spp. in Maize Grain Harvested in Portugal and Accumulation of Related Mycotoxins during Storage

**DOI:** 10.3390/foods10020375

**Published:** 2021-02-09

**Authors:** Bruna Carbas, Daniela Simões, Andreia Soares, Andreia Freitas, Bruno Ferreira, Alexandre R. F. Carvalho, Ana Sanches Silva, Tiago Pinto, Eugénio Diogo, Eugénia Andrade, Carla Brites

**Affiliations:** 1 National Institute for Agricultural and Veterinary Research (INIAV), I.P., Av. da República, Quinta do Marquês, 2780-157 Oeiras, Portugal; bruna.carbas@iniav.pt (B.C.); daniela.simoes@iniav.pt (D.S.); andreia.soares@iniav.pt (A.S.); andreia.freitas@iniav.pt (A.F.); ana.silva@iniav.pt (A.S.S.); eugenio.diogo@iniav.pt (E.D.); eugenia.andrade@iniav.pt (E.A.); 2 Centre for the Research and Technology of Agro-Environmental and Biological Sciences, University of Trás-os-Montes and Alto Douro (CITAB-UTAD), 5000-801 Vila Real, Portugal; 3 ISQ—Intelligent & Digital Systems, R&Di, Instituto de Soldadura e Qualidade, 4415-491 Grijó, Portugal; baferreira@isq.pt (B.F.); arcarvalho@isq.pt (A.R.F.C.); 4 Universidade Lusíada—Norte & COMEGI, 4760-108 Vila Nova de Famalicão, Portugal; 5 Centre for Animal Science Studies (CECA), ICETA, University of Porto, 4051-401 Porto, Portugal; 6 ANPROMIS—Associação Nacional dos Produtores de Milho e do Sorgo, Rua Mestre Lima de Freitas nº 1–5º Andar, 1549-012 Lisboa, Portugal; anpromis@anpromis.pt; 7 BioISI—Biosystems & Integrative Sciences Institute, Faculty of Sciences, University of Lisboa, 1749-016 Lisboa, Portugal; 8 GREEN-IT Bioresources for Sustainability, ITQB NOVA, Av. da República, 2780-157 Oeiras, Portugal

**Keywords:** *Zea mays* L., *Fusarium* spp., fumonisins, monitoring, CO_2_, climatic conditions

## Abstract

Maize is an important worldwide commodity susceptible to fungal contamination in the field, at harvest, and during storage. This work aimed to determine the occurrence of *Fusarium* spp. in maize grains produced in the Tagus Valley region of Portugal and the levels of related mycotoxins in the 2018 harvest and during their storage for six months in barrels, mimicking silos conditions. Continuous monitoring of temperature, CO_2_, and relative humidity levels were done, as well as the concentration of mycotoxins were evaluated and correlated with the presence of *Fusarium* spp. *F. verticillioides* was identified as the predominant *Fusarium* species. Zearalenone, deoxynivalenol and toxin T2 were not found at harvest and after storage. Maize grains showed some variability in the levels of fumonisins (Fum B1 and Fum B2). At the harvest, fumonisin B1 ranged from 1297 to 2037 µg/kg, and fumonisin B2 ranged from 411 to 618 µg/kg. Fumonisins showed a tendency to increase (20 to 40%) during six months of storage. Although a correlation between the levels of fumonisins and the monitoring parameters was not established, CO_2_ levels may be used to predict fungal activity during storage. The composition of the fungal population during storage may predict the incidence of mycotoxins.

## 1. Introduction

Maize is one of the most important agricultural products in human diets and is among the most important staple food and animal feed sources worldwide [1,2]. Maize world production is about 1.2 billion tons from around 194 million ha of land (FAOSTAT, 2020). The main producers are United States of America, China, and Brazil. In Portugal, maize has been one of the most produced cereal crop in the last years [3,4], and is the thirteenth EU-28 producer of maize with 1.4% (830 thousand tons) of total European maize production [5].

Among cereals, maize crop is the most susceptible for contamination by mycotoxigenic fungi and mycotoxins [6,7,8]. Mycotoxins are fungal secondary metabolites mainly produced by the genera *Aspergillus*, *Penicillium*, *Claviceps*, *Alternaria*, and *Fusarium*, in response to stress influenced by environmental extremes [9,10]. Fungi are also the most prevalent cause of disease in cereals [1,11,12] and are responsible for the significant decrease in yield and economic losses. *Fusarium* is one of the most important pathogenic genus affecting maize crops and includes two major fumonisin producer species, *F. verticillioides* and *F. proliferatum* [1,13]. The incidence of mycotoxins in maize grains is a main concern for human and animal health, resulting in acute or chronic consequences such as carcinogenic, teratogenic, immunosuppressive, or estrogenic effects [14]. The most significant *Fusarium* mycotoxins causing disease are fumonisins, deoxynivalenol, and zearalenone [15,16]. For better public health protection and livestock production the European Union established and harmonized the maximum limits for mycotoxins in food and foodstuffs [17] through specific regulations for all member states.

The occurrence of toxigenic fungi and mycotoxins in maize crops is influenced by climatic conditions, agronomic practices, genetic factors, fungal activity, and storage conditions [13]. Concerning climatic changes, an increase in maize sensitivity to mycotoxigenic fungiis has been anticipated with high impact on yield and economic losses. Regarding environmental conditions, fungal species prevalence and mycotoxins production have been associated with high temperature, relative humidity, and consequently, increased atmospheric CO_2_ levels before the harvest, during first drying stage and storage [18]. In vitro studies have shown that raising levels of CO_2_ around 800 ppm increase the susceptibility of maize grains to *Fusarium verticillioides* proliferation, however, the elevated CO_2_ levels decrease the fumonisins per unit fungal biomass [19]. The optimal conditions for fumonisin production are temperature around 30 °C, water activity of 0.98 a_w_ and 400 ppm CO_2_ [20]. Indeed, evaluations of temperature, water activity, and CO_2_ levels during storage of maize kernels are still limited. Therefore, post-harvest conditions, mainly during storage of maize grains in silos are crucial for the management of mycotoxin accumulation, mainly due to higher temperatures and CO_2_.

Several studies have reported mycotoxins contamination in maize-based food [7,15,21,22,23] and maize grains. In maize-based food, the levels of mycotoxins are reduced or eliminated after processing of maize flour, mainly by dilution with water and cooking [21]. However, high proportions of masked mycotoxins (mycotoxins with altered chemical structure) co-occurring with their parent forms in various cereal-based food and feedstuff can increase total exposures and pose additional health risks [24]. In addition, mycotoxin contamination of maize grains after harvest have been occurred worldwide and. Have been reported in Spain [25], South Korea [26], Republic of Serbia [27], Slovenia [7], Croatia [28], Egypt [29], Ethiopia [30], China [31], India [32], and Brazil [33], and two studies have reported the incidence of mycotoxins during storage [34,35]. In Portugal, few studies have mentioned the fungal species prevalence and mycotoxins occurrence in maize grains after harvest [36,37] and no studies have reported their accumulation during storage. Thus, due to the high toxicity of these mycotoxins and the influence of environmental conditions, the objective of this study was to investigate the occurrence of *Fusarium* species and consequent contamination of related mycotoxins in Portuguese maize grains stored for six months in barrels, monitoring CO_2_, temperature, and relative humidity parameters.

## 2. Materials and Methods

### 2.1. Sampling of Maize Grain

During the 2018 campaign, nine samples of Pioneer P0933 variety maize were collected in three different plots (JC3, JC4, and L2) from two farms, located in the Tagus Valley region of Portugal. The two farms are representative of climate and soil conditions of the Tagus Valley region, a territory with 23.000 ha of maize area that accounted for 20% of the total maize area cultivated in 2018 in Portugal. 

Plots JC3 and JC4 belong to the same farm but were subjected to different field reinforcement treatments as follows: In plot JC3, fertilization with macro- and micronutrients (N, P, and Zn) and antifungal treatment using F-BAC (EIBOL Ibérica, S. L. Valencia, Spain) applied; in plot JC4, no reinforcement treatment was applied. Plot L2 belonged to the other farm and was characterized by the application of fertilization with macronutrients (K, Mg, and S) using Patentkali, (K+S Minerals and Agriculture GmbH, Kassel, Germany). Sampling was done at three different times, i.e., at harvest (October 2018) and after 4 and 6 months of storage. Sampling at harvest was done in the field. A composite sample was prepared by mixing 3 increments collected after the harvesting of each of 6 lanes, with a total of 4 lanes per plot. 

To simulate the real storage conditions, 25 kg of maize grains, taken from the composite sample collected at harvesting time, were stored in small barrels located inside the silos. Sampling after 4 and 6 months of storage was conducted in the barrels; approximately 1 kg was collected from each barrel in each moment with a vertical probe. The sample was thoroughly mixed and divided into two subsamples as follows: One portion was used for fungal analysis, and the other portion was ground in a Retsch rotor mill (SK 300) with a sieve of trapezoid holes of 1.00 mm and stored at −20 °C for further analysis of mycotoxin quantification.

### 2.2. Chemicals Reagents

Methanol, acetonitrile (HPLC-grade), and formic acid were purchased Merck (Darmstadt, Germany). Ultrapure water used in our experiments was supplied by Milli-Q plus system from Millipore (Molsheim, France). The standards of mycotoxins (fumonisins (Fum B1 and Fum B2), zearalenone (ZEA), and toxin T2 (T2), and internal standard (zearalanone (ZAN) were purchased from Sigma-Aldrich (Madrid, Spain). 

### 2.3. Climatic Conditions

**Figure 1:**
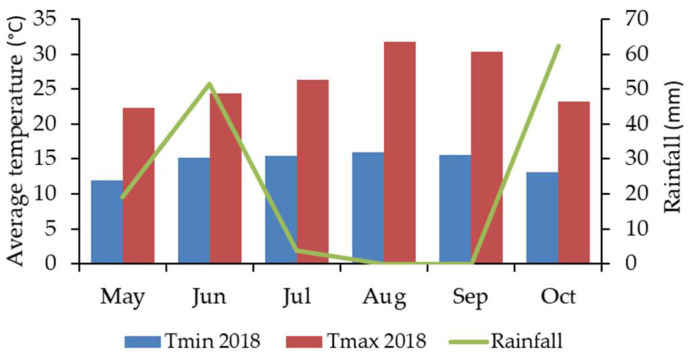
Weather station data focused on temperature (Tmin and Tmax) and rainfall in 2018, in Tagus Valley region, Portugal.

The main climate parameters related to the monthly minimum and maximum temperature, and rainfall for the 2018 season were taken from an automatic weather station, located at Lavra-Riachos with the coordinates 39°26′02.3″ N 8°29′36.0″ W. The weather condition parameters for the period of maize sowing, growing, and harvesting (May–October 2018) in Tagus Valley region are shown in Figure 1.

During vegetative cycle, the variation of Tmin was between 12.0 and 15.9 °C, Tmax between 22.3 and 31.8 °C, and rainfall between 0.0 and 62.3 mm.

### 2.4. Monitoring of Barrels

The sensors used in this analysis were TEKON’s DUOS hygrotemp and CO_2_ transmitters (Tekon Electronics, Aveiro, Portugal). The probe used could have either a CO_2_ or a temperature and humidity sensor. Probes were positioned as close as possible to the middle. The variable ranges, resolutions, and precisions are presented in Table 1. 

Therefore, when the value of 5000 ppm for CO_2_ was collected, it is implied that the value was probably greater, limited by the available sensors range. 

### 2.5. Fungal Analyses

#### 2.5.1. Sample Preparation

A fungal collection was obtained from the maize grains of this study. Fifty maize grains of each sample were surface disinfected with 1.5% NaOCl (sodium hypochlorite) for 5 min, triple rinsed with sterile distilled water, and then left to air dry in a laminar on sterile paper towels. To obtain the *Fusarium* isolates, five grains of each sample were plated onto Malachite Green Agar 2.5 [38], one in the center and one in each quadrant, with a total of 10 plates per sample. The plates were incubated for 7 days at 27 °C, for 12 h, under near UV light and 12 h darkness [39] inside the LMS Cooled Incubator, Model 410/XAL (Kent, UK), equipped with Philips TL-D 15W lights (340 to 400 nm, with peak at 355 nm). After fungal development, the maize grains contaminated with fungi morphologically compatible with the presence of *Fusarium* were counted and the *Fusarium* detected were isolated by the single spore technique [40] on Potato Dextrose Agar (PDA), BD Difco™, Fisher Scientific (Porto Salvo, Portugal) plates and incubated for 7 days under the same conditions. 

#### 2.5.2. *Fusarium* spp. Morphological Identification

To identify *Fusarium* species, macro- and microscopic (100× and 400×) morphological characteristics of all single spore isolates were observed on PDA, Carnation Leaf-piece Agar (CLA), and Spezieller Nährstoffarmer Agar (SNA) media, following Leslie and Summerell [40].

#### 2.5.3. *Fusarium* spp. Molecular Identification

The identification of the selected isolates was confirmed by DNA molecular methods. DNA of all *Fusarium* isolates was extracted following the Chelex method, as described by [41,42], using Chelex^®^ 100 Chelating Resin, analytical grade, 200–400 mesh (Bio-Rad Laboratories, Hercules, California, USA). Translation Elongation Factor 1-α (*TEF*) gene was amplified by polymerase chain reaction (PCR), using primers EF1-728F and EF1-986R [43].

The amplification reactions were performed in a Biometra TOne thermocycler (Analytik Jena GmbH, Jena, Germany) in a final volume of 20 µL of the following reaction mixture: 4 µL of 5× enzyme buffer, 1.5 µL of each primer (10 µM), 1.5 µL of dNTPs (10 mM), 2.5 µL of MgCl_2_ (25 mM), 0.3 µL of GoTaq^®^G2Flexi DNA polymerase (5 U/µL) (Promega, Germany), 5.7 µL of ultrapure water and 3 µL of DNA (independently of the concentration in ng/µL). PCR amplification consisted of an initial step at 94 °C for 5 min, followed by 39 cycles at 94 °C for 45 s, 52 °C for 30 s, and 72 °C for 90 s. A final extension step at 72 °C for 7 min was added. 

Seven microliters of each PCR reaction were analyzed in a 2% agarose gel stained with GelRed. The products were visualized and photographed under UV light (254–365 nm), (GBoxHR, Syngene, Cambridge, UK). Whenever the PCR product was of the expected size (approximately 300 bp), the remaining PCR reaction was purified with illustra™ ExoProStar™, (GE Healthcare Life Sciences, Buckinghamshire, UK) and sequenced in the Unit for Plant Breeding, INIAV, Oeiras, with the same primers as those used for the amplification. Most of the isolates were sequenced in both directions.

Nucleotide sequences were edited using the program BioEdit version 7.0.5.3 (10/28/05). After trimming the primers sequences, which could be found at the 3′ ends, the resulting sequences were compared with the sequences deposited in GenBank, NCBI (https://blast.ncbi.nlm.nih.gov/Blast.cgi (accessed on 19 December 2020)). The sequences were accepted when coverage and homology were both equal to or higher than 98%. 

### 2.6. Quantification of Mycotoxins

#### 2.6.1. Mycotoxin Extraction

The analytical procedure used to quantify the mycotoxins content of maize grains is described by Silva et al. [36]. Briefly, two grams of maize flour were weighted, 100 μL internal standard ZAN (10 μg/mL) was added, and samples extracted with 10 mL of acetonitrile 80% (*v*/*v*) for 1 h in an orbital shaker at 110 rpm (Kotterman 4010, Uetze/Hanigsen, Germany). The extracts were centrifugated at 1006 g at 5 °C for 10 min, the supernatant recovered, and the procedure was repeated once. For analysis of fumonisins, 1 mL of extract was diluted with 1 mL of ultra-pure water. For the analysis of the other mycotoxins, 8 mL of the extract was evaporated to dryness under a gentle stream of nitrogen at 40 °C. The residue was redissolved with 1 mL of acetonitrile 40% (*v*/*v*) and vortexed for 30 s. The extracts were filtered through a 0.20 µm PVDF mini-uniprep™ syringeless filters and injected into the UHPLC-ToF-MS system.

#### 2.6.2. Mycotoxin UHPLC-ToF-MS Analysis

Detection and quantification of mycotoxin were performed using the method described by Silva et al. [36]. The main *Fusarium* mycotoxin detected and quantified were fumonisins (Fum B1 and Fum B2), toxin T2 (T2), and zearalenone (ZEA). The UHPLC-ToF-MS analysis was achieved using a Nexera X2 Shimadzu UHPLC coupled with a 5600 ToF-MS detector (SCIEX, Foster City, CA, USA) equipped with a Turbo Ion Spray electrospray ionization source working in positive mode (ESI). The separation was done on a column Zorbax Eclipse Plus C18 (2.150 mm, 1.8 μm) and oven temperature was set at 30 °C. The injection volume was 20 μL of sample extract, and the flow rate was 0.5 mL/min. The mobile phase consisted of A (0.1% formic acid) and B (0.1% acetonitrile). The gradient program started with 90% of phase A at 0 min, 30% A at 12 min, 10% A at 14 min, 90% A at 15 min, and 90% A at 17 min. In terms of mass spectrometry, the acquisition was performed in full scan from 100 to 750 Da using the Analyst^®^TF (SCIEX, Foster City, CA, USA) software and with the following settings: ion source voltage of 5500 V, source temperature 575 °C, curtain gas (CUR) 30 psi, Gas 1 and Gas 2 of 55 psi, and declustering potential (DP) 100 V. Data processing was conducted using PeakView™ and MultiQuant™ (SCIEX, Foster City, CA, USA) software. The method was previously validated in our lab regarding the following parameters: determination of concentration range, linearity, limit of detection (LOD), limit of quantification, precision (repeatability and intra-laboratory reproducibility), and accuracy (using recovery assays) [36]. According to this method the LOD for Fum B1 and Fum B2 was 62.5 μg/kg, for T2 was 10 μg/kg, and for ZEA was 25 μg/kg [36].

#### 2.6.3. Deoxynivalenol (DON) Analysis

The ToF-MS method previously described is adopted in positive mode and not useful for analysis of deoxynivalenol (DON) which is determined in negative mode. Detection and semi-quantitative screening of DON in maize were performed using the method described by [44]. A biochip chemiluminescent immunoassay was previously validated and included a single extraction step with acetonitrile/methanol/water (50:40:10, *v*/*v*/*v*). The immunoassay used (Investigador™ EV 4065, Evidence Investigator Myco 7) was based on the Evidence Investigator Biochip Array technology and used a Randox Biochip i.e., a solid-state with regions containing immobilized antibodies specific to mycotoxins. According to this method the threshold and the cut-off values for 375 μg/kg of DON are, respectively, 10,894.1 and 6438.4 units of chemiluminescent signal (RLU). 

### 2.7. Statistical Analyses

The mycotoxins were measured in triplicate. The distribution of data was calculated using Test Kolmogorov–Smirnov and Shapiro–Walk with normal population distribution at *p* > 0.05 and the Levene’s test was used to evaluate the homogeneity of variance. one-way analysis of variance (ANOVA) followed by Tukey’s test, used to assess significant differences among samples. Differences were considered significant at *p* < 0.05. Pearson’s correlation coefficients were calculated to determine the relationships among fumonisin concentrations and the occurrence of *Fusarium* spp., as well as relative humidity, temperature, and CO_2_ levels. The statistical analyses applied to the analytical results were performed using SPSS Statistics 21.0 software (SPSS Inc., Chicago, IL, USA).

## 3. Results and Discussion

### 3.1. Monitoring of Maize Grain in Barrels

**Figure 2:**
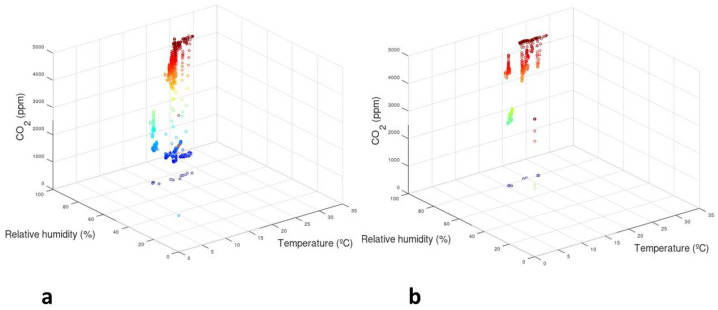
Graphical representation of relative humidity, temperature, and CO_2_ data from barrels with maize grains stored for six months. (**a**) Maize from JC3 plot; (**b**) Maize from JC4 plot. The color of the markers is determined by the CO_2_ level, from red (5000 ppm) to blue (0 ppm).

The monitoring of temperature, relative humidity, and CO_2_ parameters of JC3 and JC4 maize grains during six months of storage is summarized in Figure 2. The collected data is incomplete due to the limitation of the hardware devices themselves and other subjective reasons [45]. In the present work, data were gathered to verify if CO_2_ changed with temperature and relative humidity. In fact, most evidence involving fungal carbon metabolism involves the release of CO_2_ via cellular respiration. It has also been described that insects, in symbiosis with fungi, demonstrate preferences for certain ranges of elevated CO_2_ in which they conduct their fungal rearing [46].

The results of parameter monitored and measured values in all barrels are presented in Table 2. Due to operational constraints, monitoring of CO_2_ inside the L2 barrel was not possible, but relative humidity and temperature were registered. The mean values of relative humidity (71.33%) and temperature (16.13 °C) in the L2 barrel during six months of storage were lower than in the barrels of the other maize samples. In all cases, the level of relative humidity was considered to be high and may have promoted fungal activity. 

The levels of CO_2_ between 400 and 1000 ppm were considered within acceptable environmental variations, producing no warnings. The CO_2_ levels between 1000 and 2000 ppm were considered to produce an intermediate warning level and between 2000 and 5000 ppm were considered to require action in order to verify the CO_2_ source [20,47].

In the barrels of JC3 and JC4, high levels of CO_2_ (2860 ppm) were observed at 55.41% relative humidity and temperature near 16 °C. These levels of warnings values are easily tunable to achieve the balance between useful timely warnings and warnings overly sensitive. As the sensors provide the data in near real-time manner, useful timely warnings are desirable but warnings overly sensitive, may cause a negative impact in the users. Excessive warnings could lead to frustration for the users, diminishing the desired impact. 

Relative humidity, temperature, and CO_2_ seem to be good indicators for undesired biological activity during the cereal storage and their monitoring may be used to alert that some actions are required.

### 3.2. Fusarium spp. Presence in Maize

**Figure 3:**
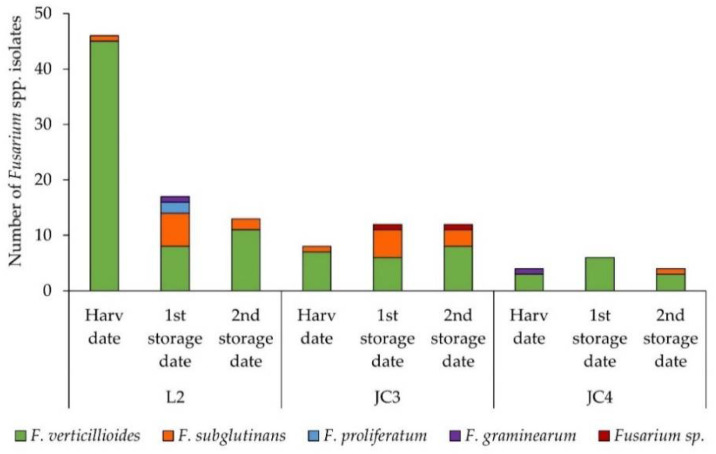
Distribution of *Fusarium* species per plot (L2, JC3, and JC4) and storage period, at harvest (Harv date), after 4 months of storage (1st storage date), and after 6 months of storage (2nd storage date) expressed as the number of contaminated grains per 50 grains analyzed.

The distributions of *Fusarium* species expressed in the number of contaminated grains per 50 grains of maize collected during harvest, at the first storage date (after 4 months of storage) and at the second storage date (after 6 months of storage) are shown in Figure 3.

The two reinforcement treatments applied in maize fields JC3 and L2 (F-BAC and Patentkali, respectively) appear to be not favorable for the control of *Fusarium* species proliferation, taking into account the higher number of *Fusarium* isolates as compared with JC4 (without any reinforcement treatment).

From the nine maize samples (total of 450 grains), 122 (27.1%) *Fusarium* spp. isolates were identified as follows: 58 (47.5%) isolates from the sample collected at the harvest date, 35 (28.7%) isolates from the sample collected at the first storage date, and 29 (23.8%) isolates from the second storage date; the most prevalent species was *F. verticillioides* (79.5%), followed by *F. subglutinans* (15.6%). On the one hand, in Poland, between 2014 and 2017, *F. verticillioides* ranged from 11.8 to 59.4% and *F. subglutinans* ranged from 0.0 to 77.2% [48]. The species *F. proliferatum* (1.6%) and *F. graminearum* (1.6%) were also isolated. Two isolates were not identifiable at the species level (Figure 3). On the other hand, in Germany, at the same period, the most frequent *Fusarium* species were *F. verticillioides* (39%), *F. graminearum* (30%), whilst *F. proliferatum* and *F. subglutinans* were detected in 13% and 2%, respectively [49]. 

The L2 samples showed a high decrease in *Fusarium* isolates from the first sampling time (during the harvest, 46 isolates) until the second one (first storage date, 17 isolates). The L2 results may be explained by the increase in species diversity and subsequent increase in competitors, as described in Ferrigo et al. [50]. In total, from L2, 76 *Fusarium* isolates were analyzed as follows: 46 (71.9%) at the harvest date, 17 (26.6%) at the first storage date, and 13 (20.3%) at the second storage date. From the samples collected at the harvest date and the second storage date, the species isolated were only *F. verticillioides* (97.8% and 84.6%, respectively) and *F. subglutinans* (2.2% and 15.4%, respectively). However, from the sample collected at the first storage date, four *Fusarium* species were isolated as follows: *F. verticillioides* (42.1%), *F. subglutinans* (35.3%), *F. proliferatum* (11.8%), and *F. graminearum* (5.9%) (Figure 3). The increase in species variability observed in L2 from the first to the second sampling dates may be due to the *Fusarium* diseases in maize being characterized by the rapid succession or co-presence of different species [13] that were not detectable in the first sampling date due to their low presence (below the limit of detection of the used method). 

Regarding JC3, 32 maize grains were contaminated with *Fusarium* species as follows: eight (25.0%) in the sample collected during harvest, and 12 (37.5%) both at the first and second storage dates. From the samples collected at the harvest date, only *F. verticillioides* (87.5%) and *F. subglutinans* (12.5%) were identified, while in the sample from the first storage date, besides these two species (50.0% and 41.7%, respectively), an unidentified *Fusarium* spp. was also detected. Similarly, from the second storage date sample, *F. verticillioides* (66.7%) and *F. subglutinans* (25.0%) were obtained, as well as an unidentified *Fusarium* spp. isolate (Figure 3).

From JC4, 14 *Fusarium* specimens were isolated as follows: four (28.6%) from both the first and last samplings, and six (42.9%) from the second sampling. At the first and last samplings, *F. verticillioides* (75.0% in both) was the predominant species with just one other species found, i.e., *F. graminearum* (25.0%) at the first, and *F. subglutinans* (25.0%) at the last sampling. From the sample collected at the first storage date, *F. verticillioides* was the only species found (Figure 3). This apparent disparity could be a sign that in the first sampling the relative amount of *F. verticillioides’* conidia were higher than the number of other species’ conidia, and therefore *F. verticillioides* could mask the other species’ presence or inhibit them. In the other samples, a succession of species could occur, not by new inoculation but because of the competition between species. During storage, the species that were not detected, at the first sampling, could proliferate, increase their presence, and be detected. As *Fusarium* diseases in maize are characterized by the co-presence and rapid succession of different species [13], this can be an explanation for the apparent increase in diversity.

These results showed a similar distribution of the *Fusarium* species in the different samples and farms, with *F. verticillioides* the predominant species isolated in maize, as has been observed worldwide in Spain [35,51], Italy [52] China [31], and India [32], followed by *F. subglutinans*, while in wheat *F. culmorun* and *F. graminearum* are more dominant. The prevalence and distribution of the *Fusarium* species depends on climatic conditions, agronomic practices, mycobiome, and the susceptibility of the plant [13]. In cooler temperate regions, *F. graminearum* has been reported to be the main species present in maize (associated with gibberella ear rot), while, in the warmer temperate regions, *Fusarium fujikuroi* complex species has mainly been reported (associated to fusarium ear rot), such as *F. verticillioides*, *F. subglutinans*, and *F. proliferatum* [53]. In this case, the results are consistent with higher temperatures (21–27 °C) detected during vegetative cycle, mainly in summer months (May to August, Figure 1). Furthermore, *F. verticillioides* and *F. subglutinans* were also the main species reported in maize in Spain [53], where climatic conditions are not that different from those in Portugal.

Regarding the number of *Fusarium* isolates collected in each plot, the following differences were found: 14 *Fusarium* specimens were isolated from JC4, 32 were isolated from JC3 (2.3 times higher than in JC4), and 76 specimens were isolated from L2 (5.4 times higher than JC4 and 2.4 times higher than JC3). On the one hand, the general conditions and mycobiomes of JC3 and JC4 were not expected to be different, given that the farm was the same and these plots were close to each other. On the other hand, the L2 plot was not close to the JC3 and JC4 farm plots, and the conditions were not necessarily the same, mainly concerning soil mycobiome. In this way, the different observations between JC3 and JC4 may be caused by the treatment. However, the differences found between L2 and JC3/4 may be explained by other factors besides the treatment, such as possible differences in soil characteristics and the use of other crops during the rotation system. 

### 3.3. Mycotoxins Analyses

**Figure 4:**
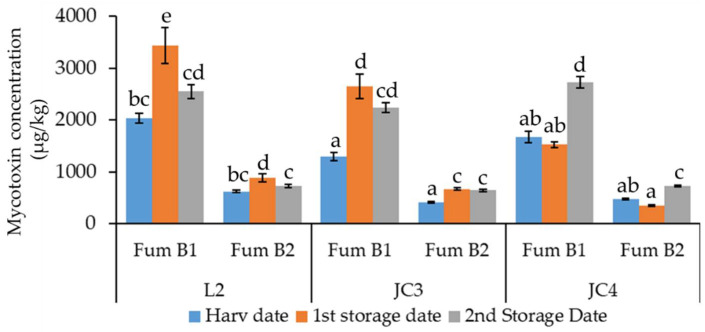
Accumulation of fumonisins in maize grain (L2, JC3, and JC4) at harvest (Harv date), four months of storage (1st storage date), and six months of storage (2nd storage date) in barrels. The absence of common letters indicates significant differences at *p <* 0.05, the Tukey’s multiple range tests were performed for each fumonisin.

Mycotoxin accumulation in maize grain stored during six months in barrels is shown in Figure 4. 

Among *Fusarium* mycotoxins analyzed on the maize grains, fumonisins were only detected, although toxin T2 (T2), zearalenone (ZEA), and deoxynivalenol (DON) were not found in the samples collected during harvest or during storage of maize grains. Similar results were obtained in maize grains collected from 2016 to 2018 in Spain, where only fumonisins were also detected [35]. Considering the maize products analyzed in Slovenia, T2 was also absent [7]; however, DON and Fum B1 were identified in maize grains from India [32] and Brazil [33], while in Serbia, Fum B1, Fum B2, ZEA, and DON were quantified in 204 maize samples [27]. The most common fumonisins found in maize was Fum B1, representing 79% of total of fumonisins, while Fum B2 represented 21%. In general, the levels of fumonisins (Fum B1 and Fum B2) increased during storage. On the harvested, the levels of Fum B1 were 2037 µg/kg for L2, 1297 µg/kg for JC3, and 1666 µg/kg for JC4. In 2006, also in Portugal, lower amounts were found in white and yellow maize (322–363 µg/kg) [37]. The levels of Fum B2 were 619, 412, and 474 µg/kg for L2, JC3 and JC4, respectively. The occurrence of mycotoxins in maize grains may be influenced by climatic conditions, as shown in Figure 1, during the vegetative cycle of maize (May to October) the temperature was from 10 to 40 °C, with higher temperatures occurring mainly between July and September and higher amounts of rainfall in the harvested month (October). The higher temperatures, verified in 2018, caused relative humidity above 85%, affecting the production of mycotoxins, mainly Fum B1 [54]. However, the mean value of Fum B1 + B2 (2168 µg/kg) found in maize grains harvested in 2018 in Portugal was lower than the level of fumonisins (3183 µg/kg) in maize from Spain in the same harvest year [25]. 

Mycotoxin accumulation in maize grains depends on the toxigenic ability of *Fusarium* species, the interaction among *Fusarium* species, and also the presence of other pests in maize before harvest [48]. In the JC4 plot, the amounts of Fum B1 and Fum B2 in maize increased from the first storage date to the second storage date, while L2 and JC3 showed the highest levels of Fum B1 and Fum B2 at the first storage date (4 months after the harvest) decreasing slightly at the second storage date. In Spain, the accumulation of Fum B1 also decreased from 509.56 to 188.42 µg/kg, and Fum B2 from 131.08 µg/kg to not detected after three months of storage in maize harvested in 2018 [35], showing an apparent faster elimination of mycotoxins in this case. In maize collected from 10 small farms in Brazil the levels of total fumonisins decreased between 1.5 and 63.9% in some farms and increased from 50 to 69% in others during storage [34]. 

The decreasing of fumonisins from first to second storage dates (mostly evident in L2) can be justified by the occurrence of biochemical reactions that induce masked mycotoxins [24] depending on the grain contamination level, temperature, and moisture, as in the case of enzymatic action [55]. Due to a relatively high incidence of *Fusarium* species in the maize grains collected, such as *F. verticillioides* which is the main producer of fumonisins, the contamination of maize with mycotoxins is a concerning issue, mainly in L2 at harvest (with 92% of maize kernels contaminated by *Fusarium* species). 

The levels of Fum B1 + B2 (4325 µg/kg) in L2, after 4 months of storage exceeded the EU limit for maize grain by 8% [17]. However, the levels of Fum B1 + Fum B2 of maize grains after six months of storage in barrels were 3277, 2883, and 3456 µg/kg, respectively, for L2, JC3, and JC4, which are values that are below the EU limit. At the end of six months of storage, the levels of fumonisins (Fum B1 and B2) were statistically similar in all maize samples. However, from harvest to the ssecond storage date, in L2 the levels of fumonisins increased by 20%, and in JC3 and JC4 the amounts of fumonisins increased by 40%. For the same period, the sensor parameters monitored in barrels recorded increases in temperature and CO_2_ levels (Table 2). 

The predominant *Fusarium* species present in L2, JC3, and JC4 samples was always *F. verticillioides* (Figure 3). As *F. verticillioides* and *F. proliferatum* are described as the main species producing fumonisins in maize grains [1], it was expected that in the samples in which these species are predominant, the levels of fumonisins were higher. This was observed in the samples collected at the harvest date, where L2 samples had a higher amount of *F. verticillioides* isolates and fumonisins than the JC3 and JC4 samples. However, this was not always observed in other cases, meaning that a high amount of *F. verticillioides* isolates does not necessarily result in higher levels of fumonisins [19,50]. Drought stress combined with high temperatures (30 °C) may also increase the incidence of mycotoxin contamination in isolates of *F. verticillioides* [20,56]. An understanding of the plant–pathogen interaction paves the way for the creation and utilization of better forms of *Fusarium* resistance in maize. Plant-derived oxylipins (oxidized lipid molecules) can mimic the physiological role of endogenous fungal oxylipins that regulate growth, development, sporogenesis, and mycotoxin biosynthesis, favoring or inhibiting these processes. In particular, oxylipins can modulate the production of fumonisins by *F. verticillioides* in maize [57]. 

Furthermore, it is such possible that the presence of other species also increases the stress on *F. verticillioides*, promoting their production of fumonisins. Nevertheless, the impact of the interactions between species on fumonisin contamination requires further investigation. On the one hand, the stress caused by the presence of other species on *F. verticillioides* may explain the increase in fumonisins observed in JC4 samples at the second storage date, due to the presence of also *F. subglutinans*, and L2 at the first storage date, due to the presence of also *F. proliferatum* and *F. subglutinans* (both fumonisin producers), and *F. graminearum*. The opposite explains the decrease in fumonisins in the sample collected from L2 at the second storage date. On the other hand, the increase in fumonisins at the first storage date from L2 and JC3 samples may also be explained by the stress caused by drying and storage of maize grains on the resilient *Fusarium* species able to produce fumonisins [13].

### 3.4. Correlations among Fumonisins, Fusarium Species, and Monitored Parameters

The correlations among fumonisins (Fum B1 and Fum B2), *Fusarium* species (*F. verticillioides*, *F. subglutinans*, *F. proliferatum*, F. graminearum, and Fusarium spp.), and monitored parameters (relative humidity, temperature, and CO_2_ levels) for harvested and stored maize grains are shown in Table 3.

Fum B1 was significantly correlated positively with Fum B2 (r = 0.96, *p* < 0.01) and *F. subglutinans* (r = 0.76, *p* < 0.01) and negatively with *F. verticillioides* (r = −0.75, *p* < 0.05). A strong correlation (r = 0.95, *p* < 0.01) beteween Fum B1 and Fum B2 was found in maize [58] and maize-based food [22]. Fum B2 was also significantly correlated positively with *F. subglutinans* (r = 0.70, *p* < 0.05) and negatively with *F. verticillioides* (r = −0.67, *p* < 0.05). These correlations highlight the greathest levels of Fum B1 and Fum B2 found in L2 and JC3 samples at the first storage date (Figure 3), due to the higher incidence of *F. subglutinans* (Figure 2). Among Fusarium species, the only strong significant correlation negatively verified was between *F. verticillioides* and *F. subglutinans* (r = −0.86, *p* < 0.01). Relative humidity was significantly correlated with CO_2_ levels (r = 0.92, *p* < 0.01).

## 4. Conclusions

Our work was the first exploratory study conducted in maize fields of the Tagus Valley region in Portugal, identifying the *Fusarium* species present in grain harvested and the levels of accumulation of fumonisins during six months of storage, while monitoring relative humidity, temperature, and CO_2_ amounts inside of stored maize grain in barrels. 

The predominant *Fusarium* species present in maize grains was always *F. verticillioides*, independent of the treatment, location (plot), or sampling time. The others *Fusarium* species found were *F. subglutinans*, *F. proliferatum*, and *F. graminearum*.

Among mycotoxins, only the fumonisins (Fum B1 and Fum B2) were identified in maize grains and always a higher incidence of Fum B1 at a ratio 4:1. Maximum amounts of fumonisins were verified after four months of storage (first storage date) in L2 and JC3 maize samples. Levels of fumonisins increased during storage periods about 20 to 40%, supported with high values of relative humidity, temperature, and CO_2_ monitored inside of barrels. 

The use of sensors for monitoring the levels of relative humidity, temperature, and CO_2_ during storage of maize grain was revealed to be a good tool to alert a possible increment of fungal activity and consequently accumulation of mycotoxins. Further research is needed on the application of good agricultural storage practices and solutions to mitigate the mycotoxin contamination in maize grain.

## Figures and Tables

**Table 1:** Sensor characteristics.

Sensor Variable	Range	Resolution	Precision
Relative humidity (%)	0–100	0.01	±2 (0–90)
Temperature (°C)	−40–80	0.01	0.1@20 °C
CO_2_ (ppm)	0–5000	1	±50 + 3% measured value

**Table 2:** Relative humidity, temperature, and CO_2_ means and ranges of the barrels of each maize stored plot (L2, JC3, and JC4) (n.m., not measured).

Plot	Storage Period	Relative Humidity (%)	Temperature (°C)	CO_2_ (ppm)
L2	First 4 months	71.2	15.2	n.m.
	Last 2 months	68.5	20.1	n.m.
	6 Months	71.3 (69.44–72.51)	16.1 (13.77–21.95)	n.m.
JC3	First 4 months	76.9	15.4	1554
	Last 2 months	75.6	20.0	3816
	6 Months	75.8 (72.87–78.93)	19.3 (14.71–23.32)	3437 (624–5000)
JC4	First 4 months	77.8	15.6	3106
	Last 2 months	80.3	20.5	4927
	6 Months	79.9 (73.25–81.23)	19.7 (14.85–24.11)	4620 (2309–5000)

**Table 3:** Pearson correlation coefficients among fumonisin levels, *Fusarium* species incidence, and monitored parameters (relative humidity, temperature, and CO_2_) from harvest and stored maize grains.

	Fum B1	Fum B2	*F. vert*	*F. subg*	*F. prolif*	*F. gram*	*F.* spp.	Rel Hum	Temp	CO_2_
Fum B1		0.96 **	−0.75 *	0.76 *	0.66	−0.16	0.17	−0.54	0.45	−0.26
Fum B2			−0.67 *	0.70 *	0.60	−0.16	0.14	−0.65	0.22	−0.43
*F. vert*				−0.86 **	−0.63	−0.16	−0.48	0.28	−0.45	0.03
*F. subg*					0.43	−0.32	0.59	−0.22	0.51	0.03
*F. prolif*						0.12	−0.19	−0.59	0.31	−0.44
*F. gram*							−0.24	0.11	−0.23	0.03
*F.* spp.								0.09	0.35	0.35
Rel. Hum									0.30	0.92 **
Temp										0.61
CO_2_										

** Significant correlation *p* < 0.01, * Significant correlation *p* < 0.05.
